# Trends in reported occupational injuries due to accidents among native-born Swedes and immigrant workers in Sweden 2003–2020

**DOI:** 10.1186/s40621-025-00581-1

**Published:** 2025-05-26

**Authors:** Emelie Thern, Alicia Nevriana, Letitia Davis, David H. Wegman, Eskil Wadensjö, Katarina Kjellberg, Theo Bodin, Devy L. Elling

**Affiliations:** 1 Unit of Occupational Medicine, Institute of Environmental Medicine, Karolinska Institutet, Stockholm, 171 77 Sweden; 2 Unit of Integrative Epidemiology, Institute of Environmental Medicine, Karolinska Institutet, Stockholm, 171 77 Sweden; 3 Massachusetts Department of Public Health, Boston, MA 02108 USA; 4 Department of Public Health, University of Massachusetts Lowell, Lowell, MA 01854 USA; 5 Swedish Institute for Social Research, Stockholm University, Stockholm, 106 91 Sweden; 6 Centre of Occupational and Environmental Medicine, Region Stockholm, Stockholm, 113 65 Sweden

**Keywords:** Occupational injuries, Immigrant workers, Time-trend analysis

## Abstract

**Background:**

Although there is a growing dependence on the immigrant workforce in many countries, recent trend analyses on the work-related health of immigrants are scarce. Thus, this study aims to fill this gap by comparing reported occupational injuries due to accidents (OIA) trends among native-born Swedes with first-generation immigrants arriving from different global regions now working in Sweden from 2003 to 2020.

**Methods:**

A repeated cross-sectional registered-based study was conducted including the total working population (18 years or older) (approximately 3.5-4 million individuals annually). Information on OIA and migrant status was obtained from nationwide registers. The incidence rate (IR) of an OIA per 1000 workers with 95% confidence intervals (CI) was calculated for region of birth, the reason for immigration, and time since immigration for each year. Joinpoint analyses were employed to detect significant shifts in the trends.

**Results:**

The IR of OIA among native-born workers demonstrated a relatively stable trend between 2003 and 2020. Immigrant workers had in general a higher IR of OIA compared to native-born workers across the study period. Among immigrant workers, a steeper downward trend in OIA was observed until 2008/2010. After 2010, the trends were relatively stable or slightly increasing, depending on the region of birth, reason for immigration, and time since immigration. The stratified analysis demonstrated varying patterns depending on sociodemographic and occupational factors.

**Conclusions:**

Immigrant workers in Sweden have a higher incidence of occupational injuries than native Swedes. Despite a general downward trend since 2003, young immigrants from Africa and the Middle East show an upward trend, highlighting a concerning increase for an already vulnerable group and potentially worsening health inequalities.

**Supplementary Information:**

The online version contains supplementary material available at 10.1186/s40621-025-00581-1.

## Background

The early 21 st century has seen significant shifts in the labor market due to globalization, economic crises, refugee crises, the COVID-19 pandemic, and digitalization. These changes have notably impacted occupational health and safety, particularly for vulnerable groups like immigrant workers (a person who is foreign-born and economically active in a host country). During The Great Recession (2007–2009), immigration increased in Europe, job insecurity increased, employment and working conditions deteriorated, and investment in occupational health and safety was reduced [1]. The great wave of migration to Europe since 2014 has resulted in an influx of immigrants and a larger variation in the region of origin and reason for migration [2]. Lastly, during the COVID-19 pandemic, many immigrant workers were overrepresented in essential and precarious jobs [3, 4]. This period also saw an increase in the differences between remote and onsite work among native-born and immigrant workers, which can have implications for the work-related health of immigrants.

Globally, there are more than 160 million immigrant workers, many of whom live and work in the European region [5]. In Sweden around 20% of the population has a foreign background, resulting in an increased share of foreign-born workers during the past several decades with the highest in 2016 [6]. Immigrant workers often have limited employment options, due to for example language barriers, recognition of qualification, and lack of professional networks [1, 7]. They are often overrepresented in manual and unskilled jobs, commonly referred to as 3D-jobs (dirty, dangerous, and demeaning) [1, 7, 8]. These jobs typically have poorer physical and psychosocial work environments [1, 7, 8]. The jobs are typically low-income and are concentrated in high-risk sectors such as construction, heavy industry, transport, services, and agriculture [9–11]. Consequently, immigrant workers generally have higher rates of adverse occupational exposures compared to native-born workers which in turn increases their risk of occupational injuries due to accidents (OIA) [1, 8, 10].

OIAs are a major public health concern and impose a significant burden on the employee, employer, and society. There is a consensus in the current literature that immigrant workers are at an increased risk of OIAs compared to native-born workers [8, 10, 12–14], which has also been echoed in Sweden [11, 15]. A limitation of previous research is, that it has grouped all immigrant workers which could mask some differences between different immigrant groups [1, 11]. Important factors such as region of birth, reason for migration, and length of stay in the host country could contribute to varying levels of vulnerability to occupational health and safety risks.

Although there is a growing dependence on the immigrant workforce in many countries, recent trend analyses on the work-related health of immigrants are scarce. Due to the labour market changes seen during the early 21 st century and evolving immigrant patterns in Sweden, it is important to examine how these factors have influenced changes in OIA to further inform public health policy. Furthermore, little attention has been paid to understanding the risk differences that arise from the heterogeneity of immigrant origins. This study aims to fill this gap by comparing reported OIA trends among native-born Swedes with first-generation immigrants arriving from different global regions now working in Sweden from 2003 to 2020. The findings could also provide valuable insights for other European countries.

## Method

### Study design and study population

This study employed a repeated cross-sectional design, examining the entire working-age population (aged 18–75 years) between 2003 and 2020. To be included in the study, individuals in the working population had to have a registered employer and an income of at least one Price Base Amount (PBA) in the year studied [16]. The PBA is calculated based on changes in general price levels by Statistics Sweden every year to determine insurance and retirement levels. The PBA ranged from approximately €3,600 to 4,800 (depending on the year) during the observation period of the present study. We used a cutoff of at least one PBA to ensure the participants of the study had been working during the year and were at risk of an OIA. The income criteria were set at a low value to include part-time workers and ensure that individuals who exited the labor market due to work-related accidents would still be included in the study. Furthermore, immigrants included in the study had to be age 18 years or older when they immigrated to Sweden. This was to ensure we did not include individuals born abroad but raised in Sweden, as they will differ from individuals migrating to Sweden later in life.

The analytical sample consisted of approximately 3.5–4 million individuals annually.

### Data source

Individuals registered as employed in Sweden were located using the Longitudinal Integrated Database for Health Insurance and Labor Market Studies (LISA), held by Statistics Sweden. The LISA register contains yearly information concerning sociodemographic characteristics, education, employment, and income for all residents aged 16 years or older [17]. To get information on migration status, the Longitudinal Database for Integration Studies (STATIV) register held by Statistics Sweden, was linked to the individuals in LISA. The STATIV register contains information such as reason for immigration, date of immigration, and region of birth [18]. Information on OIA was obtained from the Information System on Occupational Injuries (ISA) register. The Swedish Work Environment Authority is responsible for the ISA, and it contains extensive information concerning all registered OIAs.

### Variables

#### Migration status

Migration status was categorized as native-born (born in Sweden with two Swedish-born parents) and first-generation immigrants (born outside of Sweden). The first-generation immigrant group was further categorized based on region of birth (seven regions: Nordic or European Union (EU)/European Free Trade Association (EFTA), non-EU/EFTA European Countries, North America/Oceania, Africa, Asia, Middle East, Other [former Soviet Union, South America, Central America/Caribbean and unknown]). EFTA consists of Iceland, Liechtenstein, Norway and Switzerland [19]. First-generation immigrants were also categorized based on initial reason for immigration (three reasons: work, family reunification/humanitarian, other [studies, other and unknown) and time since immigration (four categories: 1–5 years, 6–10 years, 11–20 years, 20 + years). Time since immigration was calculated by subtracting the year of immigration from the year of interest (2003–2020).

Second-generation immigrants (born in Sweden with at least one foreign-born parent) were excluded from the present study as they do not have information concerning reasons for immigration and time since immigration. Also, their patterns of OIA differ slightly from both native-born Swedes and first-generation immigrants.

#### Occupational injuries due to accidents

The outcome of OIA was defined following Swedish law, such that all injuries due to accidents that occurred at the workplace or to and from work are counted as an accident [20]. In Sweden, the employer is legally responsible for reporting any OIA occurrences to the Swedish Work Environment Agency [21, 22]. If this is not done by the employer, then employees have the possibility of reporting the OIA themselves to the work environment and safety officer [22]. Although mandated by law to report OIA, there is some underreporting in the ISA register. Previous research has estimated that around 73% of the OIA in Sweden during 2013 were included in the ISA registers [21]. In the current study, we included reported OIAs (with and without sick leave that occurred at the workplace or to and from work) which were dichotomized into having an OIA and not having an OIA for each year between 2003 and 2020. Fatal occupational injuries were excluded in the analyses due to the low number (< 50 per year).

#### Covariates

In the stratified analyses, we have included information on important covariates such as sex (male/female), age (young workers < 30 years, older workers ≥ 30 years), and level of education (primary ~ 9 years, upper secondary ~ 12 years, and university ≥ 13 years) at the individual level.

We also included information on important occupational-level covariates such as the sector of economic activity (agriculture, industry, construction, commerce and hospitality, transport, financial services, public administration, education, health, other services), employer size (small to medium < 99; medium to large 100–249; large ≥ 250), ownership sector (public, private), which was defined in accordance to previous research [21].

### Statistical methods

The incidence rate (IR) per 1000 workers with 95% confidence intervals (CI) was calculated for region of birth, the reason for immigration, and time since immigration for each year. The numerator was the number of persons injured in each group of interest and the denominator was the number of person-time followed for that specific year. Individuals with more than one OIA during the same year were only counted once for that year. Individuals were censored in the analyses if they emigrated or died during that particular year.

Secondly, we utilized joinpoint analysis, an individually based method, to detect significant shifts in the trends. This method allowed us to pinpoint changes in the (log) outcome trend by applying multi-segmented regression, where the number of segments (joins) was determined using the Monte Carlo permutation method [23]. The joinpoint models identified the specific years when trend changes occurred and calculated an annual percentage change (APC) for these specific time periods. Additionally, the average APC over the whole observed study period was calculated. Bayesian Information Criterion was used for selecting the number of joinpoints, ensuring the model accurately reflects the data without overfitting.

Additional analyses were done stratified by sex, age groups, education level, employer size, and ownership sector as these factors appear to matter in relation to OIA [24]. Stratification was chosen over adjusting as we were interested in revealing patterns within specific sub-groups. Due to the low number of OIA (< 5) for some of the groups, stratified by age group, educational level, and sector, was not possible to perform for all exposures.

Data management and analyses were performed with Stata version 17 (StataCorp, College Station, TX, USA). Joinpoint Regression Program (version 4.5.0) and Microsoft Excel 365 were used for trend analysis and visualization.

## Results

Between 2003 and 2020 the total number of workers in Sweden has steadily increased as seen in Table 1, except for a slight decrease in 2020. The proportion of native-born workers has slightly decreased over the years whereas first-generation immigrant workers have increased, especially individuals born in Africa, Asia, and the Middle East. Furthermore, in the overall study population, the proportion of OIA has been steady at around 1.6% throughout the study period. During the entire study period, a total of 1 085 189 (1.61%) reported OIA were included in the current study.

**Table 1:** Prevalence of workers based on region of birth and occupational injuries among study participants between 2003–2020

Years	Total number of workers	Region of birth								Total number of OIA n (%)
		Native workers n (%)	Nordic or EU/EFTA n (%)	non-EU/EFTA countries n (%)	North America or Oceania n (%)	Africa n *(*%)	Asia n (%)	Middle East n (%)	Other n (%)	
2003	3,453,704	3,155,532 (91.4)	128,613 (3.7)	61,315 (1.8)	5,731 (0.2)	19,372 (0.6)	22,948 (0.7)	37,951 (1.1)	22,242 (3.7)	59,302 (1.7)
2004	3,469,705	3,169,077 (91.3)	128,488 (3.7)	62,022 (1.8)	5,766 (0.2)	19,443 (0.6)	24,013 (0.7)	38,508 (1.1)	22,388 (0.7)	61,320 (1.8)
2005	3,491,570	3,183,764 (91.2)	128,667 (3.7)	63,779 (1.8)	5,924 (0.2)	20,350 (0.6)	25,966 (0.7)	40,270 (1.1)	2,289 (0.7)	59,685 (1.7)
2006	3,560,686	3,234,853 (90.4)	132,061 (3.7)	67,695 (1.9)	6,214 (0.2)	22,481 (0.6)	29,147 (0.8)	44,266 (1.2)	23,969 (0.7)	59,165 (1.7)
2007	3,642,327	3,292,797 (90.4)	137,145 (3.8)	72,329 (2.0)	6,656 (0.2)	24,943 (0.7)	33,266 (0.8)	49,731 (1.4)	25,460 (0.7)	57,495 (1.6)
2008	3,676,767	3,306,884 (90.0)	140,739 (3.8)	75,709 (2.1)	7,106 (0.2)	27,427 (0.8)	37526 (0.8)	54705 (1.5)	26,671 (0.7)	58,011 (1.6)
2009	3,603,717	3,232,887 (89.7)	137,980 (3.8)	75,356 (2.1)	7,154 (0.2)	28,352 (0.8)	39,217 (0.8)	56,027 (1.6)	26,744 (0.7)	54,409 (1.5)
2010	3,635,497	3,246,868 (89.3)	140,089 (3.9)	78,546 (2.2)	7,551 (0.2)	31,057 (0.9)	43,193 (1.2)	60,630 (1.7)	27,463 (0.8)	57,542 (1.6)
2011	3,723,354	3,301,473 (88.7)	146,843 (3.9)	84,664 (2.3)	8,196 (0.2)	35,569 (1.0)	50,092 (1.4)	67,377 (1.8)	29140 (0.9)	57,689 (1.6)
2012	3,749,188	3,305,218 (88.2)	151,320 (4.0)	88,186 (2.4)	8,717 (0.2)	38,619 (1.0)	55,362 (1.5)	71,763 (1.9)	30003 (0.8)	59,517 (1.6)
2013	3,768,188	3,303,484 (87.7)	155,316 (4.1)	90,929 (2.4)	9,131 (0.2)	42,162 (1.1)	60309 (1.6)	76,397 (2.0)	30,460 (0.8)	60,615 (1.6)
2014	3,814,165	3,320,330 (87.1)	161,954 (5.3)	94,705 (2.5)	9,650 (0.3)	47,114 (1.2)	65,611 (1.6)	83,439 (2.0)	31,362 (0.8)	60,488 (1.6)
2015	3,869,381	3,340,216 (86.3)	169,368 (4.4)	99,468 (2.6)	10,218 (0.3)	53,233 (1.4)	71,275 (1.8)	92,959 (2.0)	32,644 (0.8)	61,188 (1.6)
2016	3,947,638	3,368,436 (85.3)	179,341 (4.5)	105,349 (2.7)	10,882 (0.3)	62,007 (1.6)	79,524 (1.8)	107,789 (2.0)	34,310 (0.9)	63,278 (1.6)
2017	4,012,046	3,380,543 (84.3)	188,265 (4.7)	111,688 (2.8)	11,659 (0.3)	70,702 (1.8)	88,323 (1.8)	125,275 (2.0)	35,591 (0.9)	63,725 (1.6)
2018	4,071,431	3,384,333 (83.1)	194,919 (4.8)	118,299 (2.9)	12,464 (0.3)	80,677 (2.0)	99,576 (2.5)	143,993 (3.5)	37,179 (0.9)	63,768 (1.6)
2019	4,090,118	3,365,061 (82.3)	197,583 (4.8)	123,475 (3.0)	13,030 (0.3)	85,284 (2.1)	113,188 (2.8)	154,190 (3.8)	38,307 (0.9)	67,016 (1.6)
2020	3,989,528	3,296,429 (82.6)	185,870 (4.7)	119,890 (3.0)	12,263 (0.3)	81,517 (2.1)	110,332 (2.8)	146,684 (3.7)	36,543 (0.9)	60,976 (1.5)

EU, European Union; EFTA, European Free Trade Association; OIA, Occupational injuries due to accidents

In general, a similar proportion of males and females were either native-born workers or came from other Nordic countries or the EU/EFTA (Supplementary Table 1). A larger proportion of males came from other regions except for Asia, where female workers were more prevalent. The distribution of age was quite similar across the groups, but compared to native-born workers, the level of education was higher among immigrants from North America or Oceania and generally lower among immigrants from non-EU/EFTA countries and Africa. The distribution of economic sectors was quite similar among native-born workers and immigrants, but immigrant workers from Africa and the Middle East predominantly worked in the healthcare sector. The majority of both native-born and immigrant workers were found in large companies and working in the private sector.

### Trend analysis: region of birth

**Fig. 1:**
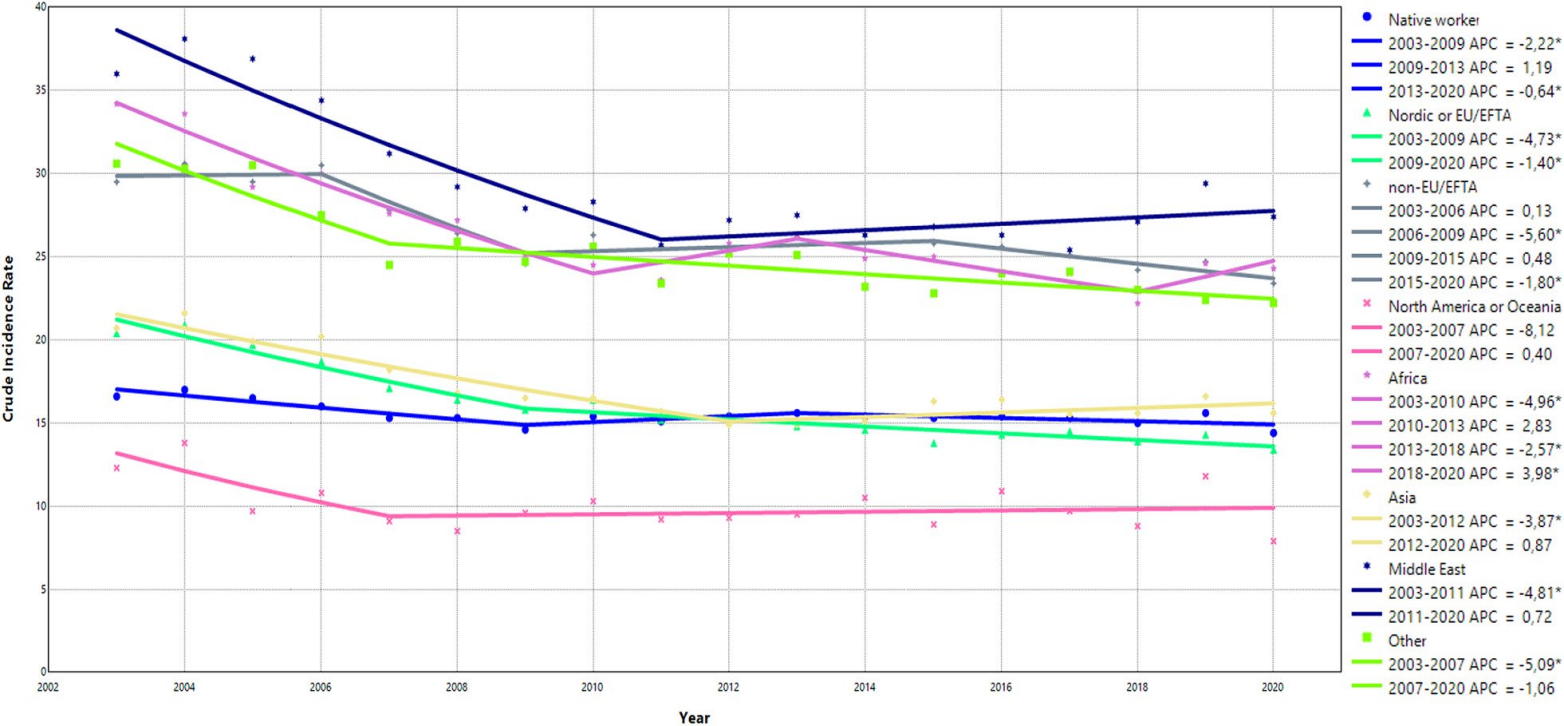
Joinpoint models fitted for region of origin. The joinpoint models identified the specific years when trend changes occurred and calculated the annual percentage change (APC) for these specific time periods. A * denotes a statistically significant change in the trends

Overall, the rate of OIA decreased over the study period when categorizing the study sample based on region of birth (Fig. 1 and Supplementary Table 2). In relation to native-born workers, immigrant workers demonstrated a higher average APC between 2003 and 2020 with the highest among workers from Nordic or EU/EFTA (2.59%).

The joinpoint analyses identified different periods with distinct trends for each of the groups. Compared to native-born workers, immigrant workers from North America or Oceania demonstrated a lower IR across all years while the remaining immigrant workers had a higher IR. All groups demonstrated a downward trend of IR of OIA from 2003 until around 2010. After 2010, most groups were quite stable with a slightly increasing trend for a few years for almost all groups (Fig. 1 and Supplement Table 3). During the last couple of years of the study period, all groups again demonstrated a general decreasing trend except for Africa, where a significant increasing trend was found (APC 3.98%, 95% CI 0.01–7.05%) between 2018 and 2020.

In the stratified analyses, the general trends were similar compared to the main analyses. In the analysis stratified by sex, native-born males and female workers had similar IR across all years while immigrant female workers, especially females born in Africa and Asia, had generally higher IR across all years compared to immigrant males (Supplementary Table 4a and 4b). Across all years, young native-born workers had a slightly higher IR than older native-born workers (≥ 30 years). These trends differ among immigrant workers, where the IR of OIA was generally slightly higher among older workers across all years and regions (Supplementary Table 5a and 5b). From 2015/2016 and onwards a steeper increase among young workers from Africa and the Middle East was found (Supplementary Table 5a). Concerning the economic sector, workers in industry, transport, and health demonstrated the highest rates among native-born workers (Supplementary Table 6b, 6e, and 6i). Similar patterns of trends but with substantially higher IR were observed among immigrants from both Africa and the Middle East (Supplementary Table 6b, 6e, and 6i), while immigrants from North America or Oceania demonstrated lower IR across all economic sectors and years compared to native-born workers (Supplementary Table 6b, 6c, 6 d, 6 h, 6i and 6j). Among native-born and immigrant workers, we found a clear trend: the larger the company, the higher the IR across all years (Supplementary Table 7a, 7b, and 7c). Consistently higher IR was demonstrated in public sectors compared to private sectors among native-born workers and immigrant workers (Supplementary Table 8a and 8b).

### Trend analysis: reason for immigration

**Fig. 2:**
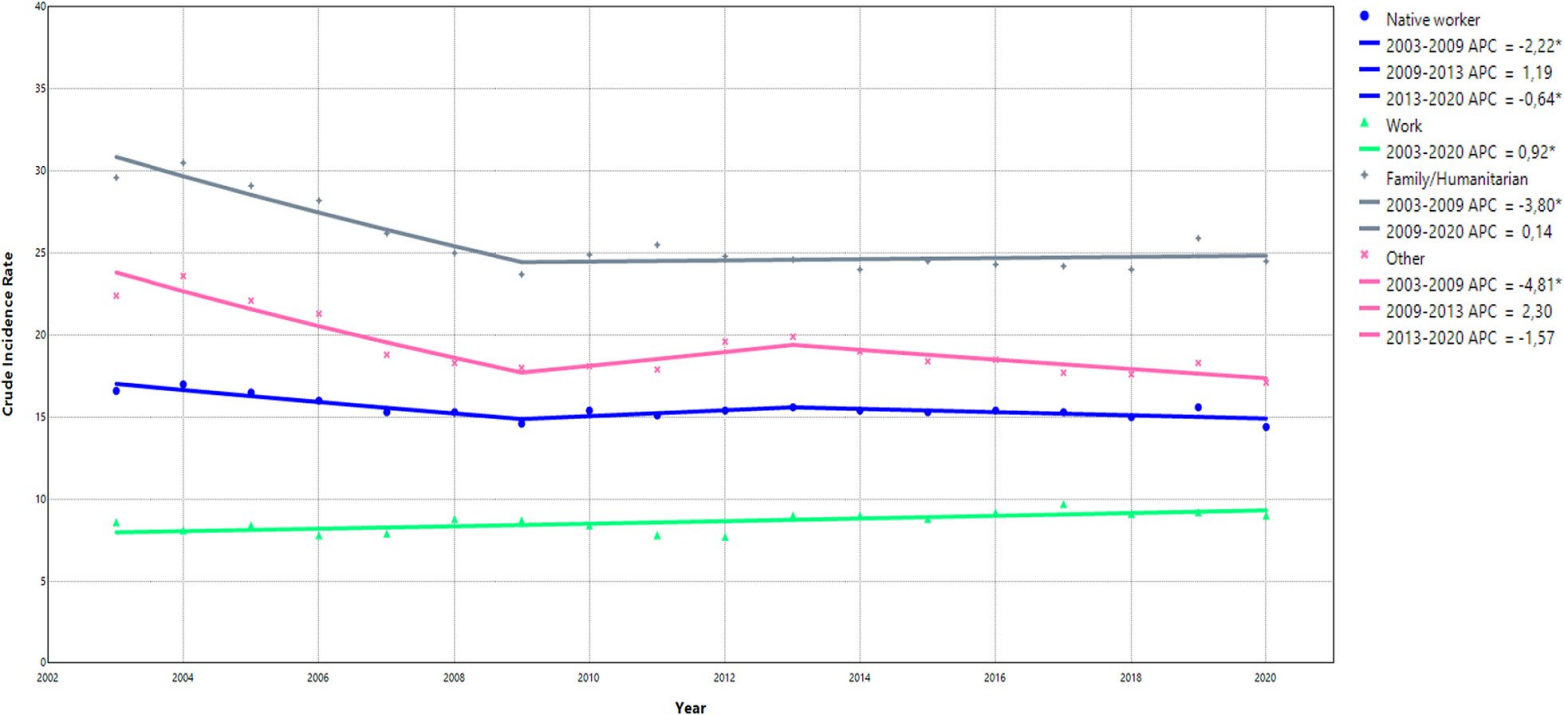
Joinpoint models fitted for reason of immigration. The joinpoint models identified the specific years when trend changes occurred and calculated the annual percentage change (APC) for these specific time periods. A * denotes a statistically significant change in the trends

Relative to native-born workers, migrants coming for work-related reasons had a consistently lower rate of OIA while migrants coming for family/humanitarian or other reasons demonstrated a consistently higher rate (Fig. 2 and Supplementary Table 9). The general declining trend of OIA across the study period was found among individuals who immigrated for family/humanitarian reasons (APC − 1.27%, 95% CI −1.65% to– 0.80%) and among individuals included in the “other” group (APC − 1.84, 95% CI −2.83% to −1.12%) (Fig. 2). Native-born workers demonstrated a generally stable (slightly decreasing) trend, while migrants coming for work-related reasons demonstrated a slightly increasing trend during the study period (APC 0.92%, 95% CI 0.44–1.65%). The largest decline in OIA rates was found between 2003 and 2009 among native-born workers and migrant workers coming for family/humanitarian or other reasons (Fig. 2 and Supplementary Table 10). Like native-born workers, immigrants coming for “other” reasons experienced a slight upward trend between 2009 and 2013, followed by a stable slightly decreasing trend.

In the additional stratified analyses, similar patterns of trends were observed as in the main analyses. Native-born male and female workers demonstrated a similar IR across all years, while females who immigrated for work, family or humanitarian reasons had generally a higher IR compared to male immigrants arriving for the same reasons (Supplementary Table 11a and 11b). Unlike native-born workers, young immigrant workers coming to Sweden for work had a higher IR than older immigrant workers at the beginning of the study period, a trend that shifted around 2010 (Supplementary Tables 12a and 12b). Among native-born workers, the IR was generally higher among workers with primary and secondary level education compared to native-born workers with university level education (Supplementary Table 13a, 13b, and 13c). In contrast, among immigrant workers, a larger difference between primary and secondary level education was found, with the highest IR observed among those with secondary level education, regardless of the reason for immigration (Supplementary Tables 13a and 13b). Among workers with university-level education, native-born workers and immigrants coming for work-related reasons had comparable IR while highly educated immigrant workers coming for family/humanitarian reasons had substantially higher IR (Supplementary Table 13c). Workers in the industry, transport, and health sectors demonstrated the highest IR of OIA (Supplementary Table 14b, 14e, and 14i). Among these workers, those who immigrated for work reasons had consistently lower IR compared to native-born workers, while immigrants who came for family/humanitarian reasons had consistently higher IR (Supplementary Table 14a to 14j). Like previous results, higher IR for OIA was found among workers working in larger companies or the public sector, which was more apparent among immigrant workers compared to native-born workers (Supplementary Tables 15a, 15b, 15c, 16a, and 16b).

### Trend analysis: time since immigration

**Fig. 3:**
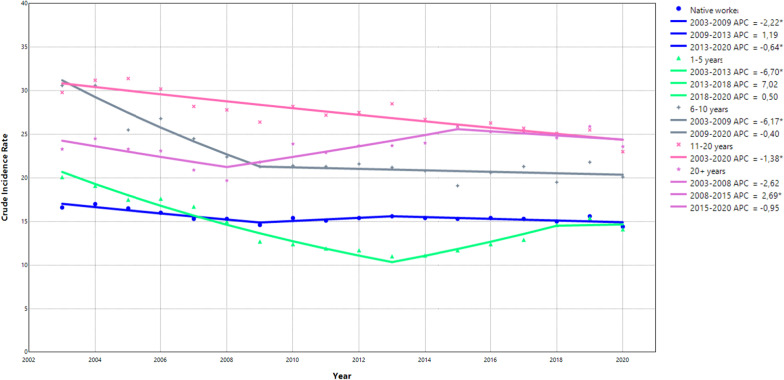
Joinpoint models fitted for time since immigration. The joinpoint models identified the specific years when trend changes occurred and calculated the annual percentage change (APC) for these specific time periods. A * denotes a statistically significant change in the trends

Overall, the rate of OIA decreased over the study period when categorizing the study sample based on time since immigration (Fig. 3 and Supplementary Table 17). Compared to native-born workers, all immigrant groups demonstrated a higher average APC between 2003 and 2020, except for immigrants who had immigrated 20 or more years ago, who showed a relatively stable trend (0.03%). After around 2008 the downward trend shifted to a slightly increasing trend for all groups except for workers that immigrated to Sweden 6–10 years ago (Fig. 3 and Supplementary Table 18).

In the stratified analyses, unlike native-born workers, immigrant female workers demonstrated a higher IR compared to immigrant males with the highest among immigrants living in Sweden for 11–20 years (Supplementary Table 19a and 19b). Immigrant workers living in Sweden for 1–5 years had similar rates of OIA as native-born Swedes across all years, regardless of educational qualification (Supplementary Table 20a, 20b, and 20c). Immigrants living in Sweden for more than 5 years demonstrated comparably higher IR compared to native-born workers (Supplementary Table 20a, 20b, and 20c). Workers in the industry, transport, and health sectors demonstrated the highest IR of OIA, where immigrants living in Sweden for 6 years or more had a higher rate of OIA compared to native-born workers (Supplementary Table 21b, 21e, and 21i). Lastly, the bigger the company the higher the IR was found among all workers regardless of time since immigration (Supplementary Table 22a, 22b, and 22c).

## Discussion

The current study aimed to identify trends of reported OIAs among native-born Swedes and immigrants working in Sweden between 2003 and 2020. Substantial differences in incidence rates were found among the different groups of immigrants working in Sweden. Overall, a downward trend was found among all groups of workers, however, the rate of this trend differed depending on the region of birth, the reason for migration as well as time since immigration. Furthermore, during the study period, a few groups especially immigrant workers from Africa and the Middle East exhibit a slight upward trend in OIA after 2008, as well as during 2019–2020.

The overall downward trend found across years and migrant exposures is in line with the reports on occupational injuries published yearly by the Swedish Work Environment Authority [25]. In Sweden, the trends of occupational injuries have historically varied greatly, especially in the 1990 s due to macro factors such as changes in the social insurance schemes [25]. A potential reason for the general decline found in the current study could be a general improvement in safety regulations and standards, as well as the technological advancements that can help mitigate risks [26]. It should also be acknowledged that immigrant workers are less likely to report OIA overall due to lack of knowledge and language barriers, especially since the OIA needs to be reported by the employee themselves in Sweden if the employer does not [21]. Additionally, less serious injuries tend to be underreported, which could have influenced the current trends as we have included all OIA irrespective of seriousness. The Great Recession of 2008 had a substantial impact on the labor market, as observed in the current study, with a leveling off from the downward trend or even an increase in the incidence rate of OIA for immigrant workers from Africa and the Middle East, as well as individuals moving for family or humanitarian reasons. During this period, many workers experienced significant employment changes [27, 28], and being newly employed is a risk factor for OIA [29, 30]. The COVID-19 pandemic is a plausible explanation for the downward trend between 2019 and 2020 that was found for most workers. During this period, non-essential workers were strongly recommended to work from home which many adhered to. As a result, the reduced time spent in the workplace likely led to fewer workplace injuries [25]. During the pandemic in Sweden, government subsidies were provided to companies to support short working hours while maintaining full pay for employees [31]. As a result, there were fewer workers present in the workplace, leading to less time at risk and potentially a decrease in occupational injuries. However, this reduction in time at work is not accurately reflected in the employment records. Immigrant workers from Africa demonstrated an increased trend in OIA during the pandemic, which could be due to being overrepresented in high-risk occupations such as health care [3, 4], which is the case in the current study population as seen in the supplementary materials.

Concerning the reason for immigration, immigrants coming for work had generally a lower OIA compared to native Swedes who are more likely to be skilled workers and have low-risk jobs [32]. Interestingly, this group of immigrants demonstrated a slight increasing trend in OIA rates throughout the study period, diverging from the general declining trend observed in all other groups. A potential reason for this could be the 2008 change to Sweden’s labor migration laws, which aimed to simplify the process of all foreign labour market migrants by transferring selection powers from the Swedish government to the employers as they understand the labour needs better. This resulted in a substantial increase in migration into low-skilled labor [33]. As a result, a significant portion of work permits have been issued for low-skilled occupations, such as in agriculture, forestry, and hospitality, where the rates of OIA are higher compared to low-risk jobs [24, 25]. Immigrant workers who originally came for family/humanitarian reasons demonstrate the highest incidence rate of OIA across all years. Immigrant workers, especially individuals coming for family/humanitarian reasons have limited employment options, due to for example language barriers, recognition of qualification, and lack of professional networks [7]. Consequently, these individuals are overrepresented in industries such as construction, agriculture, healthcare, and social services, which are all high-risk industries concerning occupational injuries [24, 25].

In the current study, we found that immigrant workers who had been living in Sweden for a longer period exhibited a higher incidence of OIA compared to recently arrived immigrants. A potential explanation for this could be that newly arriving immigrants are more likely to work part-time (i.e., fewer hours at risk of an OIA), are less likely to be unionized, and are subsequently more afraid of reporting accidents [34]. Furthermore, the higher rates of OIA found among immigrants living in Sweden for an extended period could be that they become more segregated and isolated from the native population [35, 36]. Other reasons for the higher IR among immigrants living in Sweden for an extended period could be that these immigrants have experienced upward mobility to larger workplaces, which are more likely to be unionized and have better reporting of OIA. In addition, immigrant workers change jobs at a higher frequency compared to native Swedes which could be part of the reason for the elevated incidence rate as being newly employed is a risk factor for an OIA [29, 30]. It should be acknowledged that return immigrants, who have come back to Sweden after some time away, are included in the 1–5-year group. This is because we used the most recent year of immigration to calculate the time since migration. However, these individuals might have previously lived in Sweden for an extended period, which could have influenced the results.

In the stratified analyses, we saw that trends differed between different sociodemographic sub-groups of the populations. The results of these analyses demonstrated large fluctuations between the years due to the low number of OIA in some cases, thus the shifts should be interpreted with caution. Females especially from the Middle East and Africa or females who had immigrated for family/humanitarian reasons demonstrated consistently higher IR than males coming from the same regions or reasons across all years. A potential reason for this could be the type of occupation women from the countries usually take, they are highly represented in the healthcare sector as assistant nurses and carers for the elderly which are known to be high-risk jobs [3, 4]. In line with previous reports, we found that younger native Swedes had a higher incidence rate compared to older native Swedes [25]. However, older immigrant workers generally had higher rates of OIA compared to younger workers. Compared to native workers, a substantially higher proportion of immigrant workers were classified as older workers, which could partly explain this difference. The higher rate of OIA among older workers could be due to their longer exposure to poor working conditions and the introduction of new technology in the work environment, which might be unfamiliar to the older generation of workers [37]. Young men from Africa, the Middle East, or those who immigrated for family/humanitarian reasons demonstrated a steady increase in occupational injury rates from 2015 onwards. This trend could potentially be explained by the refugee crisis, during which a substantial number of migrants, particularly young unmarried men, arrived in Sweden. Analyses stratified by the highest level of education demonstrated differences between native workers and immigrant workers across all levels of education. Previous research on immigrant workers has noted that a mismatch between qualification and occupation is common among migrant workers at all levels, which could explain these differences [32, 38]. Specifically, migrant workers with a higher level of education are more often found in low-skilled jobs where they are overqualified [32, 39]. Consequently, having highly skilled individuals in low-skilled jobs may mean they lack the motivation or knowledge to perform mundane and potentially hazardous tasks effectively. Research suggests that overqualification is more common among immigrants coming for family/humanitarian reasons compared to work reasons [32, 39], which could explain the higher incidence found among workers coming for family/humanitarian reasons.

Our results suggest that the industry, transport, and health sectors had the highest rates of OIA for all workers. However, most immigrant workers had substantially higher rates of OIA compared to native-born workers. This aligns with previous research from Italy, where the risk of OIA was found to be twice as high for immigrant workers compared to native-born workers in the same workplace [40]. This suggests that immigrants could be exposed to higher levels of hazardous work environments even within the same economic sector. In line with reports from the Swedish Work Environment Authority and previous research, our results suggest that the larger the companies the higher the incidence rate of OIA regardless of being a native-born or immigrant worker [21, 24, 25]. Potential reasons for this could be that larger companies often have more complex work environments, higher turnover, and a stronger reporting system in case of an injury [24]. The economic sector where one is employed also demonstrated significant differences in incidence rates of OIA, where a higher incidence was found among workers in the public sector compared to the private sector. In the current study, we found that the industry, transport, and health sectors were high-risk of OIA. Both the transport and health sectors include private and public entities. The work environment in the health sector differs substantially between public and private institutions, with more physically demanding and strenuous work environments found in the public sector compared to the private sector [41]. This difference could partly explain our results. Furthermore, in the public sector, more regulatory requirements are in place leading to a higher reporting of occupational injuries while the workers in the private sector might refrain from reporting due to fear of job loss or retaliation [21].

### Policy implications

Given the higher incidence of OIA among immigrant workers—especially young immigrants from Africa and the Middle East—policymakers should implement targeted occupational health and safety interventions specifically tailored to immigrant populations, such as multilingual training programs and culturally adapted educational materials. Additionally, targeted labor inspections in sectors reliant on immigrant labor such as facility management, transport and agriculture could be a step forward in risk-stratified inspection, which is under-developed in many countries, including Sweden. Finally, addressing structural inequalities by tackling precarious employment and workplace discrimination through improved contract stability and inclusive policies would further mitigate occupational risks and enhance overall health equity for immigrant workers.

### Strengths and limitations

Register-driven data including everyone in the Swedish working population for an extended period is a major strength of the current study. A further strength of the current study was the ability to include detailed information with regards to the immigrant population working, including information on the region of birth, reason, as well as time since immigration. This allows a more nuanced picture concerning the migrant workers in Sweden, as different trends emerged depending on how the study population was defined. The reason for immigration influences patterns of subsequent employment opportunities and in turn potential for exposure to safety and hazards. However, due to high levels of missing data, the region of origin is often used as a proxy to distinguish between labor and humanitarian migrants. Duration of residence is often used as a proxy for integration or acculturation into the host country, especially when other indicators of integration are unavailable [42]. The outcome of occupational injuries was well-defined and according to Swedish law, which is a strength, as it decreases the risk of misclassification bias.

Although register-based research is considered a strength in general, immigrants are an underrepresented group in the registers [43]. To be included in the register the individuals had to have resided at least one year in Sweden, consequently we miss a few of the most recent migrants coming to Sweden. In addition, since we used an income-based inclusion criterion, we missed the more vulnerable migrant workers who are on the margins of the labor market. Information on the level of education is self-reported or missing (around 2–9% missing in the current study depending on the region of birth) for immigrants in Sweden and not reported by educational institutions as is done for native Swedes, which could introduce some bias. Lastly, although the outcome is well-defined, occupational injuries need to be reported and there is an issue of underreporting especially concerning less severe occupational injuries, in certain workplaces, in smaller workplaces, and among the immigrant population [21].

## Conclusion

In conclusion, immigrants working in Sweden generally have a higher incidence rate of reported occupational injuries compared to native Swedes. While an overall downward trend has been observed across all groups of workers since 2003, immigrants from Africa and the Middle East, particularly young individuals, have shown an upward trend. This is concerning as it highlights a negative trend for a group that is already vulnerable in many ways, potentially exacerbating health inequalities. Thus, health and safety authorities should pay particular attention to injuries among immigrant workers and there are potential training opportunities for both immigrant employees and those who employ immigrants. The upward trend in OIA among those who immigrated for work reasons warrants further analysis in future research. Although some possible explanations are offered in this discussion, additional research is needed to both confirm and fully understand this phenomenon.

## Electronic supplementary material

Supplementary Material 1

## Data Availability

Availability of data and materials: The data that support the conclusions of this article are available from Statistics Sweden, but restrictions apply to the availability of these data, which were used under license for the current study, and so are not publicly available. Data are however available from the authors upon reasonable request and with permission of Statistics Sweden.

## Abbreviations

OIA: Occupational injuries due to an accident
LISA: Longitudinal Integrated Database for Health Insurance and Labor Market Studies Register
ISA: Information System on Occupational Injuries Register
STATIV: Longitudinal Database for Integration Studies Register
PBA: Price Base Amount
EEFT: European Free Trade Association
IR: Incident Rate
APC: Annual Percentage Change

## References

[1] Arici C, Ronda-Pérez E, Tamhid T, Absekava K, Porru S. Occupational health and safety of immigrant workers in Italy and Spain: A scoping review. Int J Environ Res Public Health. 2019;16(22):4416. 31718048 10.3390/ijerph16224416 PMC6888464

[2] Ackum S, Lundbäck M, Sobolevskaia T. An analysis of foreign-borns’ labor market participation (In Swedish: En analys av utrikes föddas arbetsmarknadsdeltagande). Stockholm. 2019.

[3] Benavente P, Ronda E, Diaz E. Occupation-related factors affecting the health of migrants working during the COVID-19 pandemic–a qualitative study in Norway. Int J Equity Health. 2023;22(1):220. 37848919 10.1186/s12939-023-02013-2 PMC10583456

[4] Oliva-Arocas A, Benavente P, Ronda E, Diaz E. Health of international migrant workers during the COVID-19 pandemic: a scoping review. Front Public Health. 2022;10:816597. 35252094 10.3389/fpubh.2022.816597 PMC8888537

[5] International Labour Office. ILO global estimates on international migrant workers. International Labour Office, Department of Statistics; 2021.

[6] Statistics Sweden. Foreign-born in Sweden (In Swedish: Utrikesfödda i Sverige). 2024.

[7] Herold R, Lieb M, Borho A, Voss A, Unverzagt S, Morawa E, et al. Relationship between working conditions and mental health of migrants and refugees/asylum seekers vs. natives in Europe: a systematic review. Int Arch Occup Environ Health. 2023;96(7):931–63. 37439904 10.1007/s00420-023-01981-w PMC10361874

[8] Sterud T, Tynes T, Mehlum IS, Veiersted KB, Bergbom B, Airila A, et al. A systematic review of working conditions and occupational health among immigrants in Europe and Canada. BMC Public Health. 2018;18(1):1–15. 10.1186/s12889-018-5703-3 PMC6011510 29925349

[9] Moyce SC, Schenker M. Migrant workers and their occupational health and safety. Annu Rev Public Health. 2018;39(1):351–65. 29400993 10.1146/annurev-publhealth-040617-013714

[10] Hargreaves S, Rustage K, Nellums LB, McAlpine A, Pocock N, Devakumar D, et al. Occupational health outcomes among international migrant workers: a systematic review and meta-analysis. Lancet Global Health. 2019;7(7):e872–82. 31122905 10.1016/S2214-109X(19)30204-9 PMC6565984

[11] Akay PA, Ahmadi N. The work environment of immigrant employees in sweden—a systematic review. J Int Migration Integr. 2022;23(4):2235–68.

[12] Ahonen EQ, Benavides FG, Benach J. Immigrant populations, work and health—a systematic literature review. Scand J Work Environ Health. 2007:96–104. 10.5271/sjweh.1112 17460797

[13] Kazi MR, Ferdous M, Rumana N, Vaska M, Turin TC. Injury among the immigrant population in Canada: exploring the research landscape through a systematic scoping review. Int Health. 2019;11(3):203–14. 30452624 10.1093/inthealth/ihy086

[14] Salminen S. Are immigrants at increased risk of occupational injury? A literature review. Ergon Open J. 2011;4(1).

[15] Swedish Work Environment Authority. Occupational accident risks (In Swedish: Arbetsolycksrisk). Stockholm, Sweden 2016.

[16] Statistics Sweden. Price Base amount (In Swedish: Prisbasbelopp) 2024 [Available from: https://www.scb.se/hitta-statistik/statistik-efter-amne/priser-och-konsumtion/konsumentprisindex/konsumentprisindex-kpi/pong/tabell-och-diagram/prisbasbelopp/prisbasbelopp/

[17] Statistics Sweden. Background facts, labour and education statistics, 2011:4 Intergrated database for labour market research 1990–2009 (In Swedish: Bakgrundsfakta, Arbetsmarknads- och utbildningsstatistik 2011:4, Longitudinell integrationsdatabas för Sjukförsäkrings- och Arbetsmarknadsstudier (LISA) 1990–2009). Stockholm, Sweden; 2011.

[18] Statistics Sweden. Documentation of the database STATIV year 1997–2017 (In Swedish: Dokumentation av databasen STATIV år 1997–2017) 2017.

[19] Eurostat. Glossary:European Free Trade Association (EFTA). In: Union E, editor. 2021.

[20] Swedish Work Environment Authority. Occupational accidents and work-related diseases 2022 (In Swedish: Arbetsskador 2022) 2023. Report No.: 01.

[21] Orellana C, Kreshpaj B, Burstrom B, Davis L, Frumento P, Hemmingsson T, et al. Organisational factors and under-reporting of occupational injuries in Sweden: A population-based study using capture–recapture methodology. Occup Environ Med. 2021;78(10):745–52. 33790030 10.1136/oemed-2020-107257 PMC8458053

[22] Swedish Work Environment Authority. Report occupational injury (In Swedish: Anmäl arbetsskada) 2024 [Available from: https://anmalarbetsskada.se/

[23] Kim HJ, Fay MP, Feuer EJ, Midthune DN. Permutation tests for joinpoint regression with applications to cancer rates. Stat Med. 2000;19(3):335–51. 10649300 10.1002/(sici)1097-0258(20000215)19:3<335::aid-sim336>3.0.co;2-z

[24] Swedish Work Environment Authority. Occupational accident: Factors that describe differences in risk and change over time (In Swedish: Arbetsolycksrisk Faktorer som beskriver skillnad i risk och förändring över tid). Stockholm, Sweden; 2016.

[25] Swedish Work Environment Authority. Occupational accidents and work-related diseases (In Swedish: Arbetsskador 2020). Stockholm Sweden 2020.

[26] Sidani A, Poças Martins J, Soeiro A. Catalysing construction safety: a comparative analysis of technological advancements across high-risk industries. Buildings. 2023;13(11):2885.

[27] Anxo D, Ericson T. Labour market measures in Sweden 2008–13: the crisis and beyond. Geneva: ILO; 2015.

[28] Schoon I, Bynner J. Young people and the great recession: variations in the school-to-work transition in Europe and the united States. Longitud Life Course Stud. 2019;10(2):153–73.

[29] Bena A, Giraudo M, Leombruni R, Costa G. Job tenure and work injuries: a multivariate analysis of the relation with previous experience and differences by age. BMC Public Health. 2013;13:1–9. 24053157 10.1186/1471-2458-13-869 PMC3849089

[30] Breslin FC, Dollack J, Mahood Q, Maas ET, Laberge M, Smith PM. Are new workers at elevated risk for work injury? A systematic review. Occup Environ Med. 2019;76(9):694–701. 31147382 10.1136/oemed-2018-105639

[31] MYNAK. The impact of the Covid-19 pandemic on work environments in Sweden.; 2023 Report No.: 1.

[32] Irastorza N, Bevelander P. Skilled migrants in the Swedish labour market: an analysis of employment, income and occupational status. Sustainability. 2021;13(6):3428.

[33] Irastorza N, Emilsson H, editors. The Effects of the 2008 Labour-Migration Reform in Sweden: An Analysis of Income. ECPR Joint Sessions of Workshops held at the University of Nicosia, Cyprus, April 2018; 2020.

[34] Bender G. Swedish trade unions and migration: challenges and responses. Migration and integration in a Post-Pandemic world: socioeconomic opportunities and challenges. Springer International Publishing Cham; 2023. pp. 199–231.

[35] Pega F, Govindaraj S, Tran NT. Health service use and health outcomes among international migrant workers compared with non-migrant workers: A systematic review and meta-analysis. PLoS ONE. 2021;16(6):e0252651. 34106987 10.1371/journal.pone.0252651 PMC8189512

[36] Hellman T, Brendler-Lindqvist M, Asplund S, Svartengren M. Facilitators and barriers for creating a sustainable working life for first-generation immigrants–perceptions of multiple stakeholders in Sweden. Eur J Social Work. 2024:1–14.

[37] Marsh SM, Fosbroke DE. Trends of occupational fatalities involving machines, united States, 1992–2010. Am J Ind Med. 2015;58(11):1160–73. 26358658 10.1002/ajim.22532 PMC4711279

[38] Visintin S, Tijdens K, van Klaveren M. Skill mismatch among migrant workers: evidence from a large multi-country dataset. IZA J Migration. 2015;4:1–34.

[39] Brendler-Lindqvist M, Tondel M, Helgesson M, Nordqvist T, Svartengren M. Overqualification at work and risk of hospitalization for psychiatric and somatic diseases among immigrants in Sweden–a prospective register-based study. Scand J Work Environ Health. 2022;48(8):632. 36052884 10.5271/sjweh.4055 PMC10546611

[40] Salvatore MA, Baglio G, Cacciani L, Spagnolo A, Rosano A. Work-related injuries among immigrant workers in Italy. J Immigr Minor Health. 2013;15:182–7. 22752688 10.1007/s10903-012-9673-8

[41] Heponiemi T, Kouvonen A, Sinervo T, Elovainio M. Is the public healthcare sector a more strenuous working environment than the private sector for a physician? Scand J Public Health. 2013;41(1):11–7. 23221373 10.1177/1403494812467505

[42] Schumann M, Bug M, Kajikhina K, Koschollek C, Bartig S, Lampert T, et al. The concept of acculturation in epidemiological research among migrant populations: A systematic review. SSM-population Health. 2020;10:100539. 32042888 10.1016/j.ssmph.2020.100539 PMC6997899

[43] Gauffin K. The illusion of universality: the use of nordic population registers in studies of migration, employment and health. Scand J Public Health. 2022;50(2):269–71. 32772821 10.1177/1403494820945919 PMC8873286

